# Light chain multiple myeloma, clinic features, responses to therapy and survival in a long-term study

**DOI:** 10.1186/1477-7819-12-234

**Published:** 2014-07-28

**Authors:** Jia-jia Zhang, Wan-jun Sun, Zhong-xia Huang, Shi-lun Chen, Yu-ping Zhong, Ying Hu, Na An, Men Shen, Xin Li

**Affiliations:** 1 Department of Hematology and Oncology, Beijing Chaoyang Hospital, Capital Medical University, Jingyuan Road, Beijing 100043, China; 2 Department of Hematology, The Second Artillery General Hospital, Xinwai Road, Beijing 100088, China

**Keywords:** Multiple myeloma, Light chain myeloma, Velcade, Survival

## Abstract

**Background:**

We intended to investigate the long-term clinical characteristics, responses to therapy and survival in patients with lightchain multiple myeloma (MM).

**Methods:**

Ninety-six patients were enrolled into the study. There were 42 κ-chain MM patients and 54 λ-chain MM patients. All the patients werestage III in the Durie-Salmonstaging system. Among them, 66 patients received Velcade (bortezomib) treatment and the other 30 did not.

**Results:**

The main symptoms of these patients included bone pain (77.1%), weakness and fatigue (12.5%), foamy urine (8.3%) and extramedullaryplasmocytomas (33.3%). The overall response rate (ORR) was 95.5% in patients treated with Velcade and 60%in the patients without. The median survival times were 23 months in patients treated with Velcade and 12 months in patients without. The median time of progression-free survival (PFS) was nine months in patients treated with Velcade and five months in patients without. The one-year PFS and two-year PFS were 37% and 25%, 27% and 9% for patients treated with Velcade, or without, respectively. The three-year overall survival (OS) and five-year OS were 33% and 24%, 28% and 9% for patients treated with Velcade, or without, respectively. There was no significance in OS between the two groups (*P* = 0.335). But there was significant difference in PFS between the two groups (*P* = 0.036).

**Conclusions:**

Our long-term study demonstrated that patients with lightchain myeloma appeared to have more aggressive disease courses and poor outcomes, which could be improved by treatment with Velcade.

## Background

Multiple myeloma (MM), a malignant lymphoproliferative B-cell disease characterized by the accumulation of monoclonal plasma cells in the bone marrow, is the second most frequent hematological malignancy [1,2]. The most common type of M-protein found in MM is immunoglobulin (Ig)G followed by IgA and light chain only. An exclusive production of light chains can be found in 15% of myeloma cases. Renal failure, bone disease and amyloidosis appear to be more frequent in these patients.

Lightchain multiple myeloma also appears to have a poorer prognosis than IgG and IgA subtypes when treated with chemotherapy. However, the outcomes in the light chain subtype have not been addressed specifically.

Velcade (bortezomib) is a first-in-class proteasome inhibitor, initially approved by the US Food and Drug Administration (FDA) and the European Agency for the Evaluation of Medicinal Products (EMEA) for patients with relapsed and refractory MM who have received at least two prior lines of therapy and progressed onto their last therapy [3,4]. In the present study, we conducted a long-term study (eightyears) to report the clinical characteristics, responses to therapy and survival in patients with light chain MM while they were either treated with Velcade or not.

## Methods

### Patients

Ninety-six cases of light chains multiple myeloma at Beijing Chao Yang Hospital and the Second Artillery General Hospital deriving from a series of 459 symptomatic patients with MM were included into this study from June 2005 to December 2012. There were 51 men and 45 women. The median age was 58 years (range, 28 to 86 years), and all these patients were accorded with multiple myeloma diagnostic criteria 1. These patients were staged according to International Staging System (ISS) and Durie-Salmon (DSS) staging system. Extramedullaryplasmocytomas were examined by magnetic resonance imaging (MRI) or computed tomography (CT), or were identified by the treatment effect pathology criterion with reference to the International Myeloma Working Group(IMWG).

### Clinical examination

For the examination of treatment responses, progression-free survival (PFS) and overall survival (OS), the patients were divided into two groups, the Velcade group (66 cases) and the without Velcade group (30 cases). For patients in the Velcade group, Velcade (1.0 mg/m^2^) and dexamethasone (20 mg) were given at days 1, 4, 8, 11. For patients without Velcade, they were given melphalan 8 mg/m^2^ and prednisone 60 mg/m^2^(MP)orally between days 1 and 4. Vinblastine 1.2 mg/m^2^ (M2) and simustine 20 mg/m^2^ (Me-CCUN) were given only at day 1. Also, vinblastine 0.4 mg days 1 to 4, epirubicin 10 mg days 1 to4, dexamethasone 20 mg (VAD) at days 1 to 4, 9 to12) or ifosfamide 0.5 g days 1 to 4, dexamethasone 20 mg days 1 to4, thalidomide 100 mg (CTD) each night were administered. All the patients had completed at least four cycles of Velcade or chemotherapy and had been evaluated for response to therapy after four cycles of therapy.

### Statistical analysis

The Kaplan-Meier method was used to estimate the probability of progression-free survival (PFS) and overall survival (OS). The log-rank test was used to compare PFS and OS durations among different groups. PFS was defined as the time from complete remission (CR) to relapse and progression, death from any cause, or censoring of the data on the patients. OS is defined as the time from registration to death or censoring of the data on the patients. For all other statistical analysis between Velcade and non-Velcade groups, a Student’s *t*test (SPSS version 11.0) was used. Statistical significance was determined if *P* < 0.05.

## Results

### Clinical characteristics of patients

Ninety-six patients were involved in this study, including 42 κ-chain MM patients and 54 λ-chain MM patients. The basic characteristics of the patients had no significance with the two groups (Tables 1 and 2). All the patients were stage III (DS), including 60 cases of IIIa (62.5%) and 36 cases of IIIb (37.5%). There were 12 (12.5%) patients with stage I, 26(27.1%) patients with stage II, 58 (60.4%) patients with stage III according to ISS staging (Table 3). There were 36/96 (37.5%) patients who had renal failure at the initial onset of the disease and 99/369 (27.5%) patients had renal failure of other type MM, including IgG, IgA and IgD. According to the National Kidney Foundation Practice Guidelines for Chronic Kidney Disease [5], there were 17 patients at stage 2, 12 patients at stage 3, three patients at stage 4 and four patients at stage 5. The initial symptoms included bone pain in 74cases (77.1%), weakness in 12 cases (12.5%), foamy urine ineight cases (8.3%), extramedullaryplasmocytomas in two cases (2.1%) (Table 4). During the disease progression, there were 78 (81.3%) patients had more than three areas of bone destruction, 26 (27.1%) patients complicated with pleural effusion, 32 (33.3%) patients complicated with extramedullaryplasmocytomas, 36 (37.5%) patients complicated with anemia, four (4.2%) patients complicated with hypercalcemia, one patient progressed into plasma cell leukemia and one patient complicated with M protein and skin changes (POEMS) syndrome.

**Table 1:** The characteristics of 96 light chain multiple myeloma patients

**Characteristics**	**With Velcade group(n = 66)**	**Without Velcade group(n = 30)**	**Total**	***P *** **value**
Age(years)	59(28–86)	58(43–77)	58(28–86)	>0.05
Sex(male/female)	34/29	17/16	51/45	>0.05
Albumin(g/L)	33.7(24.1-43.2)	32.1(17–42.4)	33.2(17–43.2)	>0.05
Leucocyte(X109/L)	5.4(1.0-11.7)	4.4(1.5-8.3)	5.1(1.0-11.7)	>0.05
Hemoglobin(g/L)	95.3(43–139)	99.3(51–140)	96.5(43–140)	>0.05
Platelet(X109/L)	189(33–389)	180(34–513)	186(33–513)	>0.05
C-reactive protein(mg/L)	12.76(0.62-60)	16.71(5.0-80)	13.89(0.62-80)	>0.05
Sedimentation(mm/h)	52(1.0-170)	53(2–140)	52(1–170)	>0.05
B2-microglobulin(mg/L)	7.51(1.06-38.15)	9.01(0.89-41.5)	7.96(0.89-41.5)	>0.05
Bone marrow plasmacytosis(%)	34.7(0.5-84)	40.2(3.5-95)	36.4(0.5-95)	>0.05
Lactate dehydrogenase(U/L)	179.5(91–849)	179.4(112–256)	179.5(91–849)	>0.05
Uric acid(umol/L)	346(130–590)	344(128–517)	345(128–590)	>0.05
Serum creatinine(umol/L)	177.3 (21.7-724.2)	108.5(31.27-507)	157.4(21.7-724.2)	>0.05
Light chain(g/24 hour)	23.9(1.0-128.8)	23.4(1.2-89.0)	23.8(1.2-128.8)	>0.05

**Table 2:** Clinical type of 96 light chain multiple myeloma patients

**Group type**	**κ**	**λ**	**(κ/λ)**
Velcade group(n = 66)	28	38	28/38
Without Velcade group(n = 30)	14	16	14/16
Total	42	54	42/54

**Table 3:** Durie-Salmon and International Staging System (ISS) staging of 96 light chain multiple myeloma patients

**Group subtype**	**Durie-Salmon staging**		**ISS staging**		
	**IIIA**	**IIIB**	**I**	**II**	**III**
Velcade group(n = 66)	36	30	9	17	40
Without Velcade group(n = 30)	24	6	3	9	18
Total	60	36	12	26	58

**Table 4:** The symptoms of 96 multiple myeloma patients producing light chain immunoglobulin

**Symptoms**	**Velcade group (n = 66)**	**Without Velcade group (n = 30)**	**Total**
Weakness and fatigue	10	2	12
Bone pain	51	23	74
Foam urine	4	4	8
Extramedullaryplasmocytomas	1	1	2

### Response analysis

After four cycles of treatment, the response to therapy for 96 patients were evaluated. The ORR was 95.5% (63/66), including 56.1% (37/66) CR and 39.4% (26/66) partial remission (PR) for patients treated with Velcade. The ORR was 60% (18/30) including 10% (3/30) CR and 50% (15/30) PR for the patients without Velcade treatment (Table 5).

**Table 5:** The response of 96 light chain multiple myeloma patients

**Treatment**	**Complete remission (CR)**	**Partial remission (PR)**	**Stable disease (SD)**	**Partial disease (PD)**
Velcade group (n = 66)	37(56.1%)	26(39.4%)	2(3.0%)	1(1.5%)
Without Velcade group (n = 30)	3(10%)	15(50%)	6(20%)	6(20%)

### Survival and prognosis

**Figure 1:**
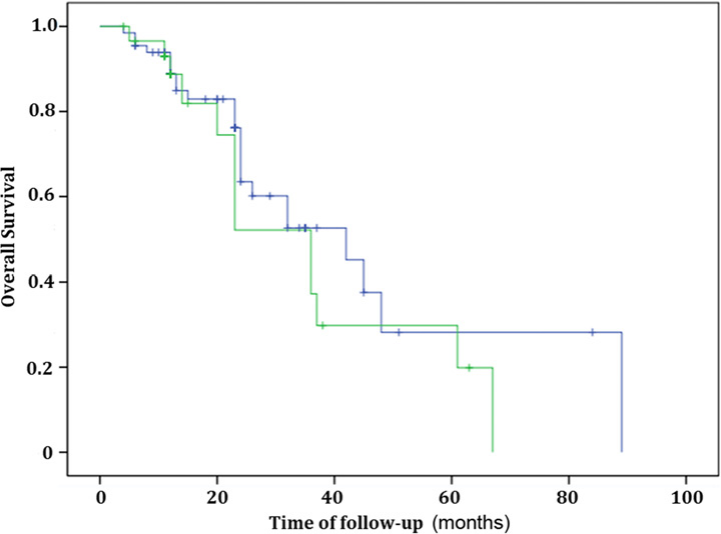
**Comparing overall survival (OS) of the light chain multiple myeloma patients in the Velcade group and the group without Velcade (*P*** **= 0.335).**

**Figure 2:**
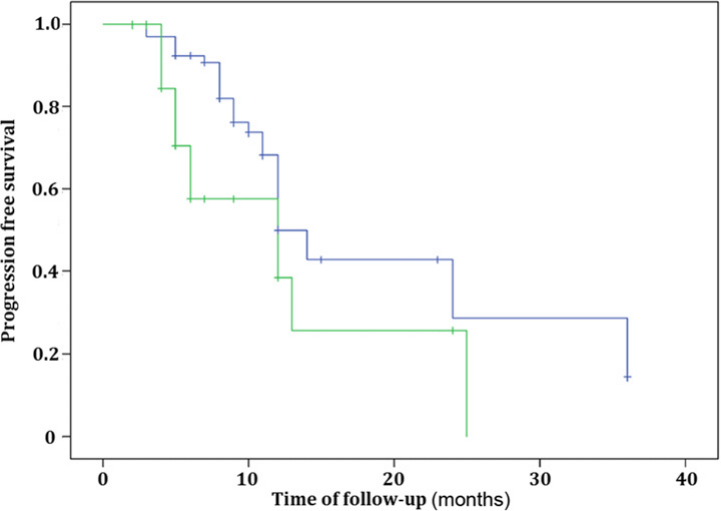
**Comparing progression-free survival (PFS) of the light chain multiple myelomapatients in the Velcade group and the group without Velcade (*P*** **= 0.036).**

At the end of the follow-up on March 31, 2013, there were 25 patients who had died in the Velcade group and 14 patients had died in the group without Velcade treatment. The median time of survival was 23 (four to 89) months in the Velcade group and 12 (four to 67) months in the group without Velcade, respectively. The median time of PFS were nine (three to 36) months for the Velcade group and five (two to 25) months for the group without Velcade, respectively. The three-year OS and five-year OS were 33% and 24%, respectively for the Velcade group, and were 28% and 9% for the group without Velcade. The one-year PFS, two-year PFS and three-year PFS were 37%, 25% and 8%, respectively for the Velcade group. The one-year PFS and two-year PFS were 27% and 9% for the group without Velcade. There was no significance with OS between two groups (*P* = 0.335) (Figure 1). However, there was significant difference in PFS between them (*P* = 0.036) (Figure 2). There were 13 patients who lived more than five years in the Velcade group, and only two patients in the group without Velcade. All the 36 patients with renal failure were treated with Velcade. Twelve patients (70.6%) with stage 2 renal functionrecovered normal kidney function, three patients (25%) with stage 3 recovered, three patients with stage 4 and four patients with stage 5did not recovernormal kidney function, and thus needed dialysis treatment.

## Discussion

In the present study, the clinical profiles of the 96 patients suggested that anemia, bone destruction, pleural effusion,extramedullaryplasmocytomas and renal failure were the most common features of light chain MM. We demonstrated that 33.3% patients had extramedullary disease, higher than the percentage reported in a previous study [6]. The difference might be explained by the fact that lightchain MM patients in our study had a high tendency of having concomitant extramedullary diseases [7-10].

## Conclusion

Overall, we found that in the patients treated with Velcade, the ORR was 95.5% (63/66), including 56.1%(37/66) CR and 39.4% (26/66) PR. This is significantly higher than the ORR of 60% (18/30) in the patients without Velcade treatment. These results are in line with previous reports [11-13], and strongly suggest the beneficial effect of Velcade on the long-term survival and prognosis for patients with light chain MM.

### Consent

Written informed consent was obtained from the patient for the publication of this report and any accompanying images.

## Abbreviations

CR: Complete remission; CT: Computed tomography; DS: Durie-Salmon staging system; Ig: Immunoglobulin; ISS: International staging system; MM: Multiple myeloma; MRI: Magnetic resonance imaging; ORR: Overall response rate; OS: Overall survival; PFS: Progression-free survival; PR: Partial remission.

## Competing interests

The authors declare that they have no competing interests.

## Author’s contribution

JZ and WS constructed the manuscript. JZ, ZH, SC and YZ collected patient’s information and conducted experiments. WS, YH, NA and MS analyzed the data. XL supervised all the procedures and approved the manuscript. All authors read and approved the final manuscript.
